# Effects of Astragalus polysaccharides on P-glycoprotein efflux pump function and protein expression in H22 hepatoma cells *in vitro*

**DOI:** 10.1186/1472-6882-12-94

**Published:** 2012-07-11

**Authors:** Qing E Tian, Huan De Li, Miao Yan, Hua-Lin Cai, Qin-You Tan, Wen-Yuan Zhang

**Affiliations:** 1 Clinical Pharmacy and Pharmacology Research Institute, The Second Xiangya Hospital, Central South University, 139 Renmin Middle Road, Changsha, Hunan, 410011, China; 2 School of Pharmaceutical Sciences, Central South University, Changsha, Hunan, 410011, China; 3 Xiangtan Central Hospital, Xiangtan, Hunan, 411100, China

**Keywords:** *Astragalus polysaccharides*, Multidrug resistance, P-glycoprotein

## Abstract

**Background:**

Astragalus polysaccharides (APS) are active constituents of *Astragalus membranaceus*. They have been widely studied, especially with respect to their immunopotentiating properties, their ability to counteract the side effects of chemotherapeutic drugs, and their anticancer properties. However, the mechanism by which APS inhibit cancer and the issue of whether that mechanism involves the reversal of multidrug resistance (MDR) is not completely clear. The present paper describes an investigation of the effects of APS on P-glycoprotein function and expression in H22 hepatoma cell lines resistant to Adriamycin (H22/ADM).

**Methods:**

H22/ADM cell lines were treated with different concentrations of APS and/or the most common chemotherapy drugs, such as Cyclophosphamid, Adriamycin, 5-Fluorouracil, Cisplatin, Etoposide, and Vincristine. Chemotherapeutic drug sensitivity, P-glycoprotein function and expression, and *MDR1* mRNA expression were detected using MTT assay, flow cytometry, Western blotting, and quantitative RT-PCR.

**Results:**

When used alone, APS had no anti-tumor activity in H22/ADM cells *in vitro*. However, it can increase the cytotoxicity of certain chemotherapy drugs, such as Cyclophosphamid, Adriamycin, 5-Fluorouracil, Cisplatin, Etoposide, and Vincristine, in H22/ADM cells. It acts in a dose-dependent manner. Compared to a blank control group, APS increased intracellular Rhodamine-123 retention and decreased P-glycoprotein efflux function in a dose-dependent manner. These factors were assessed 24 h, 48 h, and 72 h after administration. APS down regulated P-glycoprotein and *MDR1* mRNA expression in a concentration-dependent manner within a final range of 0.8–500 mg/L and in a time-dependent manner from 24–72 h.

**Conclusion:**

APS can enhance the chemosensitivity of H22/ADM cells. This may involve the downregulation of *MDR1* mRNA expression, inhibition of P-GP efflux pump function, or both, which would decrease the expression of the MDR1 protein.

## Background

Cancer is as a major public health problem worldwide [1]. The World Health Organization predicts that, by 2030, an estimated 21.4 million new cases of cancer and 13.2 million cancer deaths will occur annually [2]. Surgery, radiation, chemotherapy, and endocrine therapy are the standard cancer therapies [3]. For advanced tumors, chemotherapy is the treatment of choice. Although these drugs are effective, they are associated with severe adverse events and drug resistance, especially multidrug resistance (MDR) [4].

Drug resistance involves many mechanisms. MDR is the leading cause of treatment failure in cancer therapy. Once MDR emerges, chemotherapy becomes ineffective. High doses of drugs are required to overcome resistance, but this has toxic effects in the patient and can increase resistance further [5]. One of the underlying mechanisms of MDR is cellular overproduction of P-glycoprotein (P- GP), which acts as an efflux pump for various anticancer drugs. P-GP is encoded by the MDR1 gene. P-GP overexpression has become a therapeutic target for circumventing MDR. One strategy is to co-administer efflux pump inhibitors, but such reversal agents might increase the side effects of chemotherapy by blocking physiological anticancer drug efflux from normal cells. Although many efforts to overcome MDR have been made using first- and second-generation reversal agents, including drugs already in clinical use for other conditions (e.g. verapamil, cyclosporine A, quinidine) and analogues of first-generation drugs (e.g. dexverapamil, valspodar, cinchonine), few significant advances have been made. Clinical trials with third generation modulators (e.g. biricodar, zosuquidar, and laniquidar) specifically developed for MDR reversal are ongoing. The results, however, are not encouraging and it may be that no perfect reverser currently exists [6].

Traditional Chinese medicine (TCM) and herbal medicines in particular have been used in the treatment of cancer for thousands of years in China, Japan, South Korea, and other Asian countries. These medicines are widely accepted as current forms of complementary and alternative cancer treatments in the United States and Europe [7,8]. Experiments have shown that TCM plays an anticancer role by inducing apoptosis and differentiation, enhancing immune response, inhibiting angiogenesis, and reversing MDR [9]. TCM has great advantages in terms of increasing the sensitivity of chemo-therapeutics, reducing the side effects and complications associated with chemotherapy, and improving both patient quality of life and survival time [10]. In the search for new cancer therapeutics with low toxicity and few side effects, TCM shows promise [11].

The dried root of *Astragalus membranaceus* has a long history of medicinal use in TCM. It is traditionally prepared as a tonic that can improve the functioning of the lungs, adrenal glands, and the gastrointestinal tract, increase metabolism, promote healing, and reduce fatigue [12]. The active pharmacological constituents of *Astragalus membranaceus* include various polysaccharides, saponins, flavonoids, and L-arginine and L-canavanine [13,14]. Among these, Astragalus polysaccharides (APS) has been most widely studied, mainly with respect to its immunopotentiating properties, its ability to counteract the side effects of chemotherapeutic drugs, and its anticancer activity [12,14-24]. However, the anti-cancer mechanism of APS and the issue of whether or not it involves the reversal of multi-drug resistance are not completely clear. Reports indicate that *Astragalus membranaceus* compound preparations “Changweiqing” and “Jiexinkang” can reverse multidrug resistance and that “preventing recurrence formula for UC” can inhibit the expression of P-gp in colon tissue [25-27]. APS is the main active ingredient of *Astragalus membranaceus*, and it is worthy of further investigation.

The present study focused on investigating the effects and relevant mechanisms of APS on P-GP function and expression in H22 hepatoma cell lines resistant to Adriamycin (H22/ADM).

## Methods

### Main reagents

APS (20000–60000 mol/L) was purchased from Shanxi Undersun Biomedtech Co. Ltd.). Cyclophosphamid (MTX), Adriamycin (ADM), 5-fluorouracil (5-Fu), cisplatin (DDP), etoposide (VP-16), vincristine (VCR), verapamil (VER), and rifampicin (RFP) were purchased from the National Institutes for Food and Drug Control). A two-step immunohistochemistry detection kit PV-9000; Rhodamine123 (Rh-123); TaKaRaRNA PCRI Kit (AMV) Ver3.0; and TRIZOL reagent were purchased from SIGMA Corporation. Goat anti-mouse IgG and fluorescein-affinity pure goat anti-rabbit IgG were purchased from Jackson ImmunoResearch Laboratories. Oligonucleotides and reagents for PCR assay were purchased from SIGMA Corporation.

### Cell lines and culture conditions

H22/ADM cell lines purchased from Beijing Cowin Biotech Co. Ltd were incubated in medium containing 1 mg/L ADM to maintain its resistant characteristics. We kept H22/ADM cells in medium without ADM for 2 weeks before using these cells. The cells were cultured at 37°C in 5% CO_2_ with 100% humidity in RPMI1640 medium (Gibco/BRL, Bethesda, MD, U.S.) supplemented with 10% heat-inactivated fetal bovine serum (FBS) (Sijiqing, Hangzhou, China), 100 U/ml penicillin, 100 U/ml streptomycin.

### MTT assay for detection of H22/ADM cell proliferation

For cell growth and viability assays, 5 × 10^4^/ml H22/ADM cells were plated into 96 well cell culture plates (Costar, Charlotte, NC, U.S.) at 190 μl per well. After 24 h of incubation, they were divided into ten groups of six parallel wells each. One group contained 200 μl culture medium and served as a negative control group. Another group contained 10 μl 0.9% normal saline (NS) and served as a solvent control group. Three more groups contained DDP (0.1 mg/L, 1.0 mg/L, and 10 mg/L) and served as positive control groups. The remaining five experimental groups received different final concentrations of APS (0.8 mg/L, 4 mg/L, 20 mg/L, 100 mg/L, and 500 mg/L). After incubation for 24 h, 10 μl MTT solution (5 mg/ml) was added to each well at 37°C in the dark and allowed to incubate for at least 4 h. Formazan crystals were solubilized in 150 μl dimethyl sulfoxide (DMSO) in every well, gently swinging 10 min. The optical density (OD) was read at 540 nm and 490 nm using a plate reader (Model 550; Bio-Rad, Tokyo, Japan). Relative inhibition of cell growth was expressed as follows: Percentage (%) = (1 – [OD] test/[OD] control) × 100% [28]. Half inhibitory concentration (IC_50_) is calculated by linear regression equation. Each assay was repeated three times.

### MTT assay of the sensitivity of H22/ADM cells to chemotherapeutic drugs

For cell growth and viability assays, 5× 10^4^/ml H22/ADM cells were plated into 96 well cell culture microplates, 100 μl per well, respectively. After 6 h of incubation, the cells were divided into seven groups of six parallel wells each. One group received 200 μl culture medium. This served as a control group. The other groups received 10 μl volumes of different final concentrations of APS (0.8 mg/L, 4 mg/L, 20 mg/L, 100 mg/L, 500 mg/L). The cells that received these quantities of APS served as five experimental groups [29-31]. After incubation for 24 h, the medium was discarded and replaced with 190 μl of fresh medium per well. Then 10 μl the following concentrations of chemotherapy drugs were added into each well: ADM (0.625 μg/ml, 1.25 μg/ml, 2.5 μg/ml, 5 μg/ml, 10 μg/ml), 5-Fu (25 μg/ml, 50 μg/ml, 100 μg/ml, 200 μg/ml, 400 μg/ml), DDP (5 μg/ml, 10 μg/ml, 20 μg/ml, 40 μg/ml, 80 μg/ml), VP-16 (75 μg/ml, 150 μg/ml, 300 μg/ml, 600 μg/ml, 1200 μg/ml), VCR (1.25 μg/ml, 2.5 μg/ml, 5 μg/ml, 10 μg/ml, 20 μg/ml), CTX (100 μg/ml, 200 μg/ml, 400 μg/ml, 800 μg/ml, 1600 μg/ml). After incubation for 24 h, 10 μl MTT solution (5 mg/ml) was added into each well at 37°C in the dark for at least 4 h. Formazan crystals were solubilized in 150 μl DMSO in every well with 10 min of gentle mixing, and the OD was read at a wavelength of 540 nm and 490 nm using a plate reader. Relative inhibition of cell growth was expressed as follows: Percentage (%) = (1- [OD] test/[OD] control) × 100% [28]. IC50 was calculated using a linear regression equation. Each assay was repeated three times.

### Rh-123 accumulation assay

The efflux activity of P-GP was determined by measuring the accumulation of the fluorescent P-GP probe Rh-123 as described by Collett A et al. [32]. In brief, H22/ADM cells were incubated with RPMI1640 medium, and then RPMI1640 medium (was used as a blank control group), APS (final concentrations 0.8 mg/L, 4 mg/L, 20 mg/L, 100 mg/L, 500 mg/L), VER (P-GP antagonist, was used as positive control) 10 μmol/L, RFP (P-GP inducer, was used as positive control) 10 μmol/L for 24 h, 48 h, and 72 h before Rh-123 experiments took place. Then cells were treated with trypsin digestion, centrifugation 700 × g, 5 min, 4°C, adjusted to 1 × 10^6^/ml of cell suspension, and added to 1.5 ml EP tube, 0.5 ml/tube, Rh-123 was added to cells in the final concentration of 500 μmol/L, followed by incubation at 37°C in 5% CO_2_ incubator for 60 min. Each group included six parallel EP tubes. After this incubation period, the cells were washed twice (cold PBS, 4°C; centrifugation 700 × g, 5 min, 4°C) and fixed (cell fix, 0.5 ml). All samples were analyzed using flow cytometry.

### Western blot analysis

RFP (P-GP inducer) and VER (P-GP antagonist) were used as positive controls; H22/ADM cells were used as blank controls. Western blot analysis was performed as described previously [33]. After treatment with VER 10 μmol/L, RFP 10 μmol/L, and APS (0.8 mg/L, 4 mg/L, 20 mg/L, 100 mg/L, 500 mg/L), cells were incubated for 24 h, 48 h, or 72 h. Cells were washed twice with ice-cold PBS and total cell lysates were collected in sodium dodecyl sulfate (SDS) sample buffer (50 mM Tris–HCl, pH 6.8, 100 mM dithiothreitol (DTT), 2% SDS, 0.1% bromophenol blue, 10% glycerol). Cell lysates containing equal amounts of protein were separated by SDS-polyacrylamide gel electrophoresis (PAGE) and transferred to polyvinylidine difluoride membranes. After blocking in 5% non-fat milk in Tris-buffered saline with 0.1% Tween 20 (pH 7.6), membranes were incubated with the appropriate primary antibodies (goat anti-mouse IgG) at 4°C, overnight, and exposed to the appropriate secondary antibody (goat anti-rabbit IgG) for 3 h at 37°C. Immunoreactive proteins were visualized using the enhanced chemiluminescence system from Pierce (Rockford, IL, U.S.).

### Quantitative real time RT-PCR

RFP (P-GP inducer) and VER (P-GP antagonist) were used as positive controls; H22/ADM cells were used as blank controls. Then 5 × 10^4^/ml H22/ADM cells were seeded and incubated for 6 h until adherent. The cells were treated with VER 10 μmol/L, RFP 10 μmol/L, and APS (0.8 mg/L, 4 mg/L, 20 mg/L, 100 mg/L, 500 mg/L) and then incubated for 24 h, 48 h, or 72 h. Each set of exposure conditions for mRNA analysis was reproduced and confirmed by two additional independent experiments, representing biological triplicates.

*MDR1* mRNA expression in H22/ADM cells was detected by quantitative RT-PCR. Total RNA was extracted using the TRIZOL reagent according to the manufacturer’s instructions and reverse-transcribed to cDNA using a Gene Amp RNA PCR kit in a DNA thermal cycler (Bio-Rad). QRT-PCR was performed with SYBR green PCR master mix in an ABI Prism 7700 real time PCR machine (Applied Biosystems, Foster City, CA, U.S.). The synthesized cDNA served as a template in a (25 μL) reaction. A non-template control was included in all experiments. Primer sequences are as follows: *P-GP*, sense: 5′-TAA TGC GAC AGG AGA TAG GCT-3′, and antisense: 5′-CCG CCA TTG ACT GAA AGA ACA T-3′; *GAPDH*: sense: 5′-GAG TCA ACG GAT TTG GTC G-3′, and antisense: 5′-CGG AAG ATG GTG ATG GGA TT-3′. QRT-PCR was performed at 94°C for 4 min, followed by 40 cycles at 94°C for 15 s, 60°C for 25 s, and 72°C for 25 s. Data were analyzed with sequence detector software (v1.9, Applied Biosystems). The mean Ct value for duplicate measurements was used to detect the expression of the target gene with normalization to a housekeeping gene used as an internal control (glyceraldehyde-3-phosphate dehydrogenase GAPDH) according to the 2 − ΔCt formula.

### Statistical analysis

Statistical analysis for the data of cell cytotoxicity, P-GP function and expression assays were performed on SPSS 14.0 software (v14, SPSS Inc. Chicago, IL, U.S.). The differences in variables between the groups were analyzed by one-way ANOVA. Real-time PCR data were analyzed using the SDS software on the ABI PRISM®7700 sequence detection system. The confidence limit was set at 95%. Values of *P* < 0.05 were considered statistically significant.

## Results

### Effects of APS on H22/ADM cell proliferation

Table 1 shows that the inhibition rates of APS ranged from 1.11% to 62.40% in a concentration-dependent manner. However, the maximum inhibition rate was only 62.40%. The IC50 value of APS was 251.77 mg/L, which is significantly higher than the positive control DDP [IC50 = 0.04 mg/L] (*P* <0.05).

**Table 1:** Impact of APS on H22/ADM cell proliferation (n = 6)

**Agents**	**Concentration (mg/L)**	**OD Value (mean ± SD)**	**Inhibition Rate (%)**	**IC50 (mg/L)**	**IC50 95%CI (mg/L)**
APS	0.8	0.71 ± 0.15	1.11		
	4	0.67 ± 0.12	6.69		
	20	0.61 ± 0.09	15.04	251.77^★^	1.6 × 10–1.2 × 10^3^
	100	0.47 ± 0.04	34.54		
	500	0.27 ± 0.14	62.40		
DDP	0.1	0.29 ± 0.02	59.61		
	1.0	0.21 ± 0.04	70.75	0.04	1.7 × 10^-3^–0.74
	10	0.06 ± 0.03	91.64		
NS		1.018 ± 1.06	ND		
DMSO	0.1	0.918 ± 0.134	ND		

^★^*P* < 0.05, vs. DDP; ND: Not calculated; SD: Standard deviation; NS: normal saline.

### MTT assay of sensitivity of chemotherapeutic drugs

**Figure 1:**
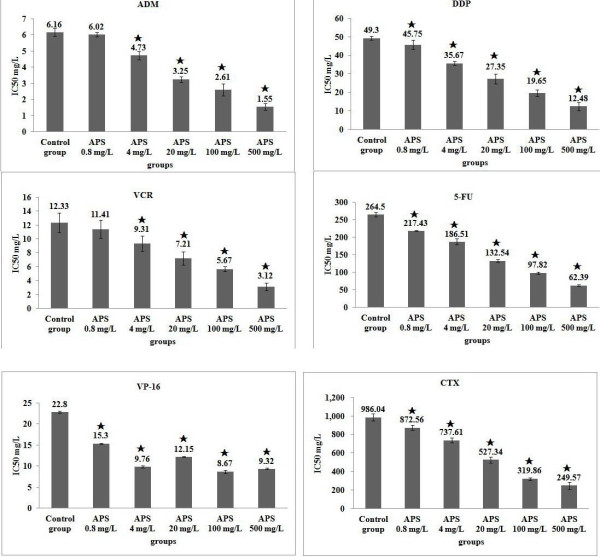
**IC50 of APS combined with ADM, DDP, VCR, 5-FU, VP-16 or CTX in H22/ADM cells.**^★^*P* < 0.05, vs. control group.

The IC50 of different concentrations of APS combined with chemotherapeutic drugs (ADM, 5-Fu, DDP, VP-16, VCR, or CTX) and the control group (ADM, 5-Fu, DDP, VP-16, VCR, or CTX, when applied alone) are shown in Figure 1. The difference between APS combined with ADM or VCR and the control group was not significant at APS 0.8 mg/L, but APS combined with ADM or VCR could was found to significantly reduce the IC50 value (*P* < 0.05, VS. control group) at APS concentrations of 4–500 mg/L; APS combined with 5-Fu, DDP, VP-16, or CTX was found to significantly reduce the IC50 value (*P* < 0.05, VS. control group) at APS concentrations of 0.8–500 mg/L. For APS combined with chemotherapeutic drugs (ADM, 5-Fu, DDP, VCR, or CTX), the IC50 value decreased with increasing concentrations of APS within the range of 0.8–500 mg/L, and for APS combined with VP-16, the IC50 value decreased trend with increasing concentrations of APS was not obvious.

### Rh-123 accumulation assay

**Figure 2:**
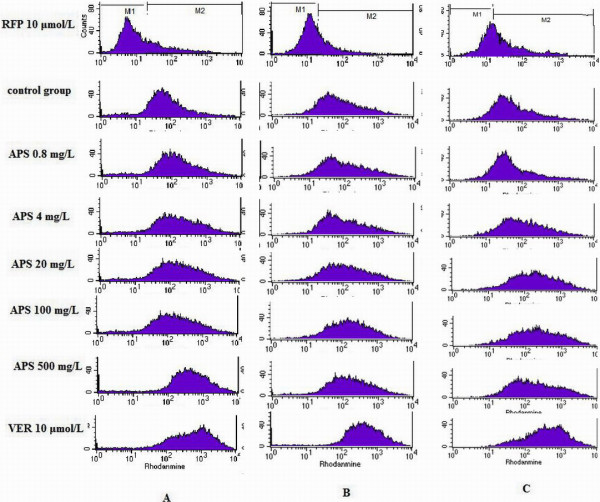
**Flow cytometry analysis of P-gp activity with Rh-123 in H22/ADM cells. A: Rh-123 fluorescence intensity curve in H22/ADM cells after exposure to chemotherapeutic agents at 24 h. B**: Rh-123 fluorescence intensity curve in H22/ADM cells after exposure to chemotherapy agents at 48 h. **C**: Rh-123 fluorescence intensity curve in H22/ADM cells after exposure to chemotherapy agents at 72 h.

Rh-123 retention was measured to evaluate the P-GP transport activity in the H22/ADM cells. The fluorescent dye Rh-123 is a substrate of P-GP, and its cellular retention has been shown to reflect P-GP function. Rh-123 efflux was measured by counting cells in the M1 region of the plot. The marker bar M1 was set to indicate cells with high Rh-123 efflux. Marker bar M2 was set to indicate the cells with low Rh-123 efflux. As shown in Figure 2, compared with the control group, the RFP group fluorescence curve shifted to the left, suggesting that intracellular Rh-123 uptake decreased and P-GP efflux increased; the fluorescence curves of the APS (APS 0.8 mg/L, APS 4 mg/L, APS 20 mg/L, APS 100 mg/L, APS 500 mg/L) and VRE groups shifted to the right, suggesting that intracellular Rh-123 retention increased and P-GP efflux function decreased over the 24 h, 48 h, and 72 h time periods. However, this was only a general trend, no shifts were obvious for the RFP group or the 0.8 mg/L at 72 h.

Table 2 shows the intracellular fluorescence intensity of Rh-123 accumulation in H22/ADM cells after exposure to chemotherapeutic agents at 24 h, 48 h, and 72 h. The intensity of the intracellular fluorescence of Rh-123 was lower in RFP 10 μmol/L group than in the control group, and the intensity of the intracellular fluorescence of Rh-123 was higher in VRE and APS groups than in the control group at 24 h, 48 h, and 72 h. The intensity of the intracellular fluorescence of Rh-123 increased with increasing concentrations of APS within the range of 0.8–500 mg/L. The difference between the APS 0.8 mg/L group and the control group was not significant at 72 h, but other APS groups saw significant increases (*P* < 0.05, VS. control group) in the intensity of intracellular fluorescence of Rh-123 at 24 h, 48 h, and 72 h. The results show that P-GP efflux activity was inhibited by APS.

**Table 2:** Intracellular fluorescence intensity of Rh-123 accumulation in H22/ADM cells (n = 6)

**Group**	**Fluorescence intensity (mean ± SD)**		
	**24 h**	**48 h**	**72 h**
RFP 10 μmol/L	87.49 ± 1.26^**★**^	133.71 ± 1.59^▴^	131.41 ± 1.54
Control group	114.77 ± 1.16	198.51 ± 0.69	136.18 ± 1.32
APS 0.8 mg/L	135.52 ± 0.92^**★**^	255.14 ± 0.61^▴^	137.40 ± 2.01
APS 4 mg/L	257.38 ± 0.97^**★**^	266.32 ± 2.03^▴^	250.32 ± 1.80^●^
APS 20 mg/L	357.34 ± 0.80^**★**^	285.75 ± 1.30^▴^	367.35 ± 1.31^●^
APS 100 mg/L	342.83 ± 0.94^**★**^	307.42 ± 1.12^▴^	460.04 ± 1.74^●^
APS 500 mg/L	701.30 ± 1.14^**★**^	345.27 ± 1.84^▴^	458.43 ± 0.63^●^
VER 10 μmol/L	928.04 ± 1.17^**★**^	706.04 ± 2.02^▴^	657.91 ± 1.28^●^

^**★**^*P* < 0.05, vs. Control group (24 h); ^▴^*P* < 0.05, vs. Control group (48 h); ^●^*P* < 0.05, vs. Control group (72 h).

### Western blot analysis H22/ADM cell P-GP expression

**Figure 3:**
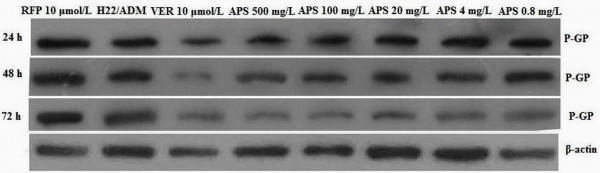
P-GP expressions in H22/ADM cells after exposure to the different chemotherapy agents at different time points (24 h, 48 h, and 72 h).

The protein levels of P-GP in H22/ADM cell lines were detected by Western blotting. As indicated in Figure 3, the level of P-GP protein was lower in the APS (APS 0.8 mg/L, APS 4 mg/L, APS 20 mg/L, APS 100 mg/L, APS 500 mg/L) and VER groups than in the H22/ADM group at 24 h, 48 h, and 72 h. The protein level of P-GP was higher in the RFP group compared to H22/ADM group at 24 h, 48 h, and 72 h time points. The same concentration of APS affected P-GP expression in different ways at different times. P-GP expression was largest at 24 h and then fell in a time-dependent manner. Different concentrations of APS were found to down regulate P-GP expression with increasing concentrations of APS in a concentration-dependent manner in the range of 0.8–500 mg/L. These results showed that APS could down regulate P-GP expression in a concentration-dependent manner within the range of 0.8–500 mg/L and in a time-dependent manner from 24 h to 72 h.

### Quantitative RT-PCR detection of *MDR1* mRNA in H22/ADM cells

After intervention for 24 h, 48 h, and 72 h, the levels of *MDR1* mRNA expression in H22/ADM cells were detected by quantitative RT-PCR. As indicated in Table 3, the levels of *MDR1* mRNA expression decreased (*P* < 0.05) in APS (APS 0.8 mg/L, APS 4 mg/L, APS 20 mg/L, APS 100 mg/L, APS 500 mg/L) and VER groups compared to H22/ADM group at 24 h, 48 h, and 72 h time points; the levels of *MDR1* mRNA expression were higher in the RFP group than in the H22/ADM group at 24 h, 48 h and 72 h. *MDR1* mRNA expression decreased with increasing concentrations of APS within the range of 0.8–500 mg/L.

**Table 3:** ***MDR1*mRNA expression in H22/ADM cells at different times (24 h, 48 h, 72 h) (n = 6)**

**Group**	**2-▽▽Ct (Mean ± SD)**		
	**24 h**	**48 h**	**72 h**
RFP 10 μmol/L	1.670 ± 0.027	1.977 ± 0.064	2.726 ± 0.086
H22/ADM	1.525 ± 0.039	1.797 ± 0.108	2.563 ± 0.048
VER 10 μmol/L	0.519 ± 0.045^**★**^	0.497 ± 0.107^▴^	0.440 ± 0.114^●^
APS 500 mg/L	0.616 ± 0.035^**★**^	0.565 ± 0.033^▴^	0.496 ± 0.072^●^
APS 100 mg/L	0.792 ± 0.163^**★**^	0.742 ± 0.077^▴^	0.898 ± 0.042^●^
APS 20 mg/L	0.890 ± 0.055^**★**^	1.067 ± 0.107^▴^	1.076 ± 0.108^●^
APS 4 mg/L	1.128 ± 0.051^**★**^	1.183 ± 0.058^▴^	1.412 ± 0.027^●^
APS 0.8 mg/L	1.260 ± 0.113^**★**^	1.406 ± 0.024^▴^	1.736 ± 0.063^●^

^**★**^*P* < 0.05, vs. H22/ADM group (24 h); ^▴^*P* < 0.05, vs. H22/ADM group (48 h);^●^*P* < 0.05, vs. H22/ADM group (72 h).

Existing data on the relationship between mRNA and protein levels create a somewhat contradictory picture, as shown in Figure 2 and Table 3. For example, there is no correlation between time dependent changes in the *MDR1* mRNA levels and corresponding P-GP levels. This merits further study.

## Discussion

The dried root of *Astragalus membranaceus* has a long history of medicinal use in TCM. It is an adjunct anticancer agent and it has been the subject of a great deal of research [17,22,24]. Studies have shown that APS has anti-tumor activity *in vitro* when applied alone in certain tumor cell lines, such as murine renal cell carcinoma, murine bladder tumors, HepG2 cells, human gastric cancer SCG-7901 cells, human colon cancer cell lines, hormone-sensitive (MCF-7) breast cancer cell lines, and human hepatocellular carcinoma [13,17,24,25,29,30]. Animal tumor models and clinical studies have also confirmed that APS has anti-tumor activity [16,21-23,34]. However, there have only been a few reports of the treatment of drug-resistant tumor cells with APS. The present study shows at a final concentration range of 0.8–500 mg/L, the IC50 value of APS for H22/ADM cell proliferation was 251.77 mg/L. According to National Cancer Institute guidelines, extracts with IC50 values < 20 μg/ml are considered active *in vitro*[35]. The results show that that APS has no anti-tumor activity for H22/ADM cells *in vitro* when applied alone. However, patients with advanced cancer can be treated with APS combined with chemotherapeutic drugs. It has been found to inhibit tumor development, decrease the toxic-adverse effects of chemotherapy, elevate immune function, and improve patient quality of life [34-36]. For example, Guo et al. reported that treatment with APS injections integrated with vinorelbine and cisplatin significantly improved quality of life in patients with advanced non-small-cell lung cancer over vinorelbine and cisplatin alone [23]. Animal tumor models and *in vitro* studies confirmed that APS can enhance the chemo-sensitivity of the chemotherapy drugs for non-drug-resistant tumor cells [37-39]. For example, Li et al. reported that the weight of tumors in subjects treated with APS and ADM was significantly lower than those of the NS group [16]. Cui R. et al. reported that hepatocarcinogenesis could be prevented in rats fed with the aqueous extract of Astragalus, which is mainly composed of Astragalus polysaccharides [21]. For H22/ADM resistant cells, as shown in Figure 1 that APS combined with ADM or VCR could significantly reduce the IC50 value (*P* < 0.05, VS. control group) at APS concentration range of 4 mg/L to 500 mg/L; APS combined with 5-Fu, DDP, VP-16, or CTX could significantly reduce the IC50 value (*P* < 0.05, VS. control group) at APS concentration range of 0.8–500 mg/L. In this way, APS can enhance the chemo-sensitivity of the most common chemotherapy drugs *in vitro*. The present results were partially supported by the results of the above-mentioned studies.

Those research teams speculated that the anti-tumor activity of APS might involve enhancement of immune function and induction of apoptosis. However, the mechanism underlying these effects remains to be determined. Changweiqing (Radix Astragali, Radix Codonopsis, Rhizoma Atraety lodis macroce phalae) was found to reverse the drug resistance of colon cancer cells by reducing the expression of MDR1/P-GP [25]. APS is the main active ingredient of Radix Astragali, its involvement in the reversal of MDR merits further investigation [13,14].

Drug resistance in tumor cells has been shown to be related to MDR1 and P-GP overexpression [40-42]. In the present study, APS was found to enhance the chemo-sensitivity of H22/ADM cell lines to certain drugs. To determine whether APS is involved in P-GP expression and/or its efflux function, the P-GP inducer RFP and P-GP inhibitor VER were used as positive controls, and an H22/ADM group was used as a blank control.

Rh-123 is a cationic dye. It has been used extensively as a marker of P-GP-mediated transport in both *in vitro* and *in vivo* studies [43-47]. In the present study, the intracellular fluorescence intensity of Rh-123 increased with increasing concentrations of APS in a concentration-dependent manner in the range of 0.8–500 mg/L. The results show that P-GP efflux activity was inhibited by APS.

Western blot analysis of P-GP expression and quantitative RT-PCR detection of *MDR1* mRNA expression in H22/ADM cell lines showed that APS reduced P-GP protein expression and *MDR1* mRNA expression in a concentration-dependent manner within the range of 0.8–500 mg/L and in time-dependent manner from 24 h to 72 h. APS not only inhibited P-GP efflux but also reduced P-GP and *MDR1* mRNA expression in a concentration-dependent manner. However, this effect was not uniform across all times. For example, APS inhibition of P-GP efflux function did not occur in a time-dependent manner; and there was no visible correlation between time-dependent changes in the MDR1 mRNA levels and corresponding P-GP levels. The modest correlation between mRNA expression and protein abundance in large-scale data sets can be explained in part by experimental challenges, such as technological limitations, and in part by fundamental biological factors in the transcription and translation processes. Translation is a complicated biological process, and many of the details still merit further investigation. For example, highly expressed proteins may not necessarily require large quantities of mRNA if they have higher than average translation rates. This merits further study [48].

Drug resistance is a major obstacle to the successful treatment of cancer. Tumor cells either fail to reduce in size following chemotherapy or cancer recurs. The phenomenon of MDR is particularly problematic because it involves the simultaneous resistance to numerous chemotherapeutics of different classes, and the mechanism by which tumors develop MDR is very complex, P-GP overexpression is one important factor in this process [49]. Studies have shown that APS has anti-tumor activity in certain tumor cell lines in vitro and in animal models of certain tumors. The present study confirmed that APS can downregulate *MDR1* mRNA expression, inhibit P-GP efflux function and decrease its expression, thereby increasing the intracellular concentration of chemotherapeutic drugs. This may be the mechanism behind its secondary anti-cancer effects.

It has been reported that APS can increase the sensitivity of chemotherapeutics, reducing the side effects and complications associated with chemotherapy, and improve patient quality of life and survival time [12,14,19,20,23]. In this way, in the search for new cancer therapeutics with minimal toxicity and few side effects, APS is a promising candidate.

## Conclusion

In summary, APS was found to enhance the chemosensitivity of the H22/ADM cell line, which may be related to downregulation of *MDR1* mRNA expression and inhibition of P-GP efflux pump function, which decreases its MDR1 protein expression.

## Abbreviations

MDR: Multidrug resistance; H22/ADM: H22 hepatoma resistant to adriamycin cell lines; P- GP: P-glycoprotein; TCM: Traditional Chinese medicine; APS: Astragalus polysaccharides; MTX: Cyclophosphamid; ADM: Adriamycin; 5-Fu: 5-fluorouracil; DDP: Cisplatin; VP-16: Etoposide; VCR: Vincristine; VER: Verapamil; RFP: Rifampicin; NS: Normal salin; DMSO: DimeThyl sulfoxide; OD: Optical density; IC_50_: Half inhibitory concentration; GAPDH: Glyceraldehyde-3-phosphate dehydrogenase.

## Competing interests

The authors declare that they have no competing interests.

## Authors’ contributions

QET has made substantial contributions to conception and design, acquisition of data, analysis and interpretation of data, and drafted the manuscript; HDL has made substantial contributions to conception and design; MY, H-LC, Q-YT and W-YZ have been involved in drafting the manuscript and revising it critically for important intellectual content. All authors read and approved the final manuscript.

## Pre-publication history

The pre-publication history for this paper can be accessed here:

http://www.biomedcentral.com/1472-6882/12/94/prepub
